# Content of Trace Elements and Human Health Risk Assessment via Consumption of Commercially Important Fishes from Montenegrin Coast

**DOI:** 10.3390/foods12040762

**Published:** 2023-02-09

**Authors:** Neda Bošković, Danijela Joksimović, Oliver Bajt

**Affiliations:** 1 Institute of Marine Biology, University of Montenegro, Put I Bokeljške Brigade 68, 85330 Kotor, Montenegro; 2 National Institute of Biology, Marine Biology Station, Fornače 41, 6330 Piran, Slovenia; 3 Faculty of Maritime Studies and Transport, University of Ljubljana, Pot pomorscakov 4, 6320 Portoroz, Slovenia

**Keywords:** trace elements, *Mullus barbatus*, *Merluccius merluccius*, human health risk assessment, Montenegro, Adriatic Sea

## Abstract

Muscle tissues of *Mullus barbatus* and *Merluccius merluccius* were analyzed for the presence of selected trace elements (As, Hg, Cd, and Pb) to determine the value of the daily intake of trace elements from fish consumption and to assess the risk to human health. The mean concentrations of As in the muscle tissue of *M. barbatus* and *M. merluccius* for the entire period were 19.689 mg/kg wet weight (ww) and 8.356 mg/kg ww, Hg 0.497 mg/kg ww and 0.153 mg/kg ww, and Pb 0.031 mg/kg ww and 0.025 mg/kg ww, respectively. The concentrations of Cd in all fish sampled were below the detection limit (<0.02 mg/kg ww). The evaluation of the potential health risk assessments based on the target hazard quotient (THQ) and estimated daily intake (EDI) indicated that the intake of As in both fish species and Hg for *M. barbatus* could pose an appreciable risk to human health. The calculated hazard index (HI) was higher than 1 for both fish species. The continuous monitoring of trace elements’ concentrations in fish is strongly recommended, as the results demonstrate potential health risks due to the presence of As and Hg.

## 1. Introduction

The increasing presence of various pollutants in the marine environments poses a global threat to marine organisms. Trace elements (TEs) are natural elements present in the environment or introduced by different human activities [1]. They are persistent, and their bioaccumulation/biomagnification in the food chain has been studied by various authors [2,3,4,5]. Some TEs are of biogenic origin, but their increased concentration in the environment is usually the result of various anthropogenic activities [1]. Fish is an important source of many essential nutrients such as vitamins, minerals, proteins, and unsaturated fatty acids (omega-3) [3,4].

On the other hand, humans may suffer adverse effects from consuming fish contaminated with TEs such as arsenic (As), mercury (Hg), lead (Pb), and cadmium (Cd) [4]. TEs accumulated in fish may disrupt the beneficial nutritional values of fish and reach humans through the food chain, leading to health risks [2]. Fish accumulate TEs through food, water, and sediment. The accumulation of TEs in fish tissues varies depending on the concentration of the TE in the environment, the natural habitat of fish, feeding habits, age, trophic level, duration of exposure, as well as on the individual TE and the rate of its absorption, deposition, and excretion [1,4,6,7]. The effects of toxic TEs disrupt various metabolic processes, especially in the early stages of fish development. These adverse effects can lead to developmental delays, morphological and functional deformities, or the death of the most susceptible individuals [8].

Many fish species are regarded as good bioindicators of aquatic environment pollutants and represent a good monitoring tool to assess changes in the environment [9,10,11], because they accumulate organic and inorganic pollutants well and are good indicators of long-term effects [12]. Human exposure to arsenic (As), mercury (Hg), cadmium (Cd), and lead (Pb) elements are among the top ten chemicals of public health concern [13].

European hake (*Merluccius merluccius*) and red mullet (*Mullus barbatus*) were selected as bioindicators of TE pollution due to their widespread distribution and consumer and economic importance off the coast of Montenegro. The red mullet, a benthic fish, was recognized by the MedPol program (Mediterranean Pollution Monitoring and Research Programme) as an important indicator species for pollution monitoring [14], while the European hake, a benthopelagic demersal fish, was recommended as a bioindicator species for TEs [15]. Habitat, age, gender, body mass, and feeding habits of fish, as well as the geographic region, temperature, salinity, and the pH value of the surrounding water, may influence the concentration, bioaccumulation, and biomagnification of contaminant components [16]. Environmental factors such as the nature of sediments, temperature, salinity, and the seasonal cycle of absorption/dissolution of elements in certain areas may influence the seasonal variability in TEs’ concentration and TEs’ bioaccumulation by marine organisms [17,18].

Montenegro occupies the south-eastern end of the Adriatic Sea. The water body of the Adriatic Sea is recognized as highly dynamic and ecologically diverse. Large rivers such as Vjosë, Bojana, Drin, Neretva, Krka, Soča, and Po, flowing into the Adriatic Sea, are significant transporters of industrial and domestic waste to coastal and marine waters [19,20]. TE pollution in the Montenegrin marine environment may derive from natural phenomena and also be a result of anthropogenic activities, including industrial processes, industrial and domestic waste, sewage discharges, the presence of commercial ports, and agricultural activities [21,22].

The objective of the present study was to investigate, for the first time, concentrations of As, Pb, Cd, and Hg in two commercially important fish species: European hake (*M. merluccius*) and the red mullet (*M. barbatus*). Additionally, the objective of the present study was to assess the health risk to humans on the Montenegrin coast of the Southern Adriatic from the consumption of fish. The results of this study will provide important information about the presence of TEs in fish species and health risk for humans related to TE exposure from the consumption of the studied fish species. In addition, the results of this study will serve as a basis for future research and analysis.

## 2. Materials and Methods

### 2.1. Sampling

**Figure 1:**
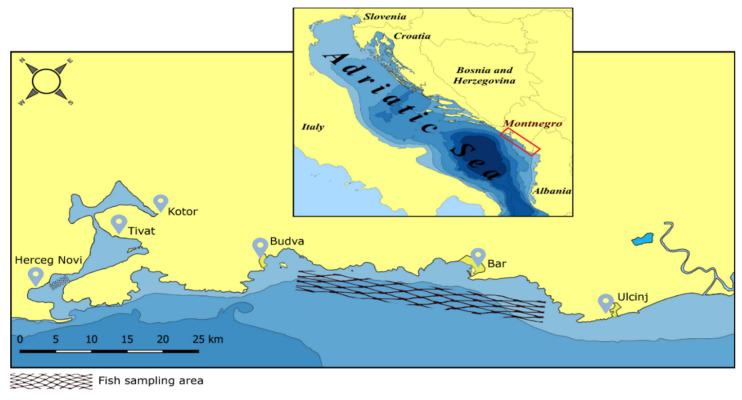
Map of the studied area.

*M. barbatus* and *M. merluccius* were sampled in two areas with different geographical and ecological features and different levels of anthropogenic activities along the coast of Montenegro in the southern part of the Adriatic Sea (Figure 1).

Fish samples were caught with triple set nets in Boka Kotorska Bay and demersal nets in the coastal part of the open sea in water depths ranging from 50 to 160 m. Samples were collected in the spring and autumn of 2019–2020. Ten fish (commercial size) were collected from each area and sampling period. The collected fish samples were taken to the laboratory, where the weight (g) and length (mm) of the studied fish species were measured. The weight of the studied *M. barbatus* and *M. merluccius* ranged from 130.2 g to 183.3 g (mean 158.7 ± 18.2 g) and from 247 g to 311.5 g (mean 270.7 ± 20.2 g), respectively, while the length of the studied *M. barbatus* and *M. merluccius* ranged from 35.3 mm to 72.5 mm (mean 60.4 ± 24.3 mm) and from 177.4 mm to 321.7 mm (mean 220.5 ± 50.4 mm), respectively.

### 2.2. Trace Element Analyzes

Fish samples were washed with Milli-Q water and dissected to separate the muscle tissues. The skin, bones, and internal components were removed and discarded, while the muscle tissues were homogenized. To decompose the organic matter in the samples and dissolve all TEs, the homogenized muscle tissues of the fish (1 g) were digested in a microwave oven (Speedwave Xpert, Berghof, Germany) with 5 mL of HNO_3_ (>68%, PrimarPlus—trace analysis grade, Fisher Chemical, Göteborg, Sweden) and 2 mL of H_2_O_2_ (>30%, analytical reagent grade, Fisher Chemical, Göteborg, Sweden). In each digestion, alongside the sample blank, the certified reference materials (CRMs) of International Atomic Energy Agency (IAEA) 407 and 436 were digested and analyzed. After digestion, the cuvettes were cooled to room temperature and the contents were transferred quantitatively to polypropylene bottles. Arsenic (As) concentrations in red mullet and European hake muscle tissues were determined using inductively coupled plasma optical emission spectrometry (ICP-OES), with a Thermo iCAP 7400 Duo 7400 device. Mercury (Hg) in fish samples was measured using a direct mercury analyzer (DMA), the Advanced Mercury Analyzer AMA 254 (Altec, Leco, St. Joseph, MI, USA), while cadmium (Cd) and lead (Pb) were measured using graphite furnace atomic absorption spectrometry (GF-AAS) with the Shimadzu AA 6800 device. All analytical data were subject to strict quality control. The instruments were calibrated with the calibration standards. To increase the accuracy of the analytical methods, all samples were analyzed in duplicate. The analytical limits of detection (LODs) (mg/kg) were 0.009048 for As, 0.000382 for Cd, 0.001619 for Pb, and 0.000205 for Hg. Method recovery was evaluated using CRMs and ranged between 98.9 and 100.4% for all elements (Table 1). TE concentrations in fish muscle tissues were determined in mg/kg wet weight (ww). Table 1 shows the comparison of certified and measured concentrations of TEs in fish samples in CRMs.

The preparation of samples, as well as the analysis of TEs in muscle tissue of fish, was carried out in the laboratory of the Centre for Ecotoxicological Research of Podgorica (Podgorica, Montenegro) according to standard methods MEST EN 14084:2009 and according to the laboratory manual of the IAEA agency, Monaco Marine Ecosystem Laboratory. In order to reduce the possibility of contamination, the laboratory utensils used were prewashed with 10% (*v*/*v*) HNO_3_.

### 2.3. Risk Assessment for Human Health

#### 2.3.1. Target Hazard Quotient (THQ)

To determine the risk to human health from the consumption of the studied fish contaminated with TEs, the Target Hazard Quotient (THQ) was calculated. The THQ represents the ratio between exposure and the reference dose (RfDo) [23]. When the exposure level is greater than the RfDo, the THQ is above 1, indicating that the exposed population is at health risk from the ingestion of an individual TE through the consumption of the studied fish [24]. The THQ is expressed by the following equation [25]:

(1) $$\mathrm{THQ}=\frac{\mathrm{EF}\times \mathrm{ED}\;\times \;\mathrm{MS}\;\times \;C}{\mathrm{RfDo}\;\times \;\mathrm{BW}\;\times \;\mathrm{AT}}\;⨯\;10^{-3}$$

where EF—an exposure frequency (365 days/year); ED—an exposure duration—average human lifetime (70 years); MS—a fish meal size (17.5 g/day) [26]; C—the average TE concentration of each group of fish species (mg/kg ww); RfDo—the estimated oral reference dose of the TEs (mg/kg of body weight per day) provided by the USEPA [27]. RfDo values: As 0.0003 mg/kg∙day; Hg 0.0001 mg/kg∙day (assuming that Hg measured in fish was integrally in its methylated form and Cd 0.0001 mg/kg∙day); BW—body weight, adult (70 kg); AT—an averaging exposure time (EF × ED).

As there is no evidence of a threshold below which it is harmless, the intake of Pb could be allowed, but the USEPA declined to set an RfDo for Pb. The THQ for Pb was calculated using the equation [28,29]:

(2) $$\mathrm{THQ}=\frac{\;C}{\mathrm{ML}}$$

where C—the average TE concentration of each group of fish species (mg/kg ww); ML—1.5 mg/kg ww for Pb is the maximum permitted level of the contaminant (Maximum Regulation Limit) set by the Regulation (EC) No 1881/2006 [30], amended by the Regulation (EU) 2015/1005 [31].

#### 2.3.2. Hazard Index (HI)

The hazard index (HI) was used to evaluate the potential risk assessment with associated combined or interactive effects [32,33] triggered by TEs collectively present in the studied fish. When the value of HI is above 1, there might be a concern for potential health effects [25]. HI was determined by summing all the calculated THQ values for the specific elements [25]:

(3) $$\mathrm{HI}=\overset{n}{\underset{i=1}{\sum }}\mathrm{THQi}$$

where THQi—the target hazard quotient of a single element; n—the number of examined elements (in the present study *n* = 4).

#### 2.3.3. Estimated Daily Intake (EDI)

The Estimated Daily Intake (EDI) per fish meal was determined by multiplying the mean concentrations of each TE by the amount of fish consumed per day for a 70 kg adult. Based on USEPA guidance [34], it was assumed that cooking has no effect on contaminants and that the ingested dose was equal to the adsorbed contaminant dose. The EDI was determined using the following equation [26]:

(4) $$\mathrm{EDI}=\frac{C\;\times \;\mathrm{MS}}{\mathrm{BW}}$$

where C—the average TE concentration of each group of fish species (mg/kg ww); MS—a fish meal size (17.5 g/day) [26]; BW—a body weight, adult (70 kg).

### 2.4. Statistical Analyses

For statistical analysis, data were square root transformed based on Bray–Curtis similarity matrices before analysis. To demonstrate the distribution and similarity of the TE concentration in fish species based on the different sampling areas studied, seasons, and for both years, a principal coordinate analysis (PCO) was performed. The Monte Carlo test (PERMANOVA) was used to determine if there were any significant differences among the studied species, studied areas, and seasons. All statistical significance was reported for *p* < 0.05. PRIMER 7 software with the PERMANOVA+ add-on package (Albany, New Zealand) [35] was used for all statistical analyses.

## 3. Results

### 3.1. Concentrations of TEs in Fish

The concentrations of studied TEs in muscle tissues of *M. barbatus* and *M. merluccius* sampled in two areas and in two different seasons of the year are presented in Table 2. Considering all the studied TEs in both fish species, sampling areas, and sampling periods of this study, the obtained mean values were in the order of As > Hg > Pb > Cd.

The concentrations of As, Hg, and Pb were higher in the muscle tissues of *M. barbatus* during the entire research period in relation to the concentrations of the same TEs in the muscle tissues of *M. merluccius*, while the concentrations of Cd in all fish sampled were below the detection limit (Table 2).

As was the most abundant element in the muscle tissue of *M. barbatus* and *M. merluccius*. The concentration of As in the muscle tissue of *M. barbatus* ranged from 8.572 to 39.501 mg/kg ww, and in the muscle tissue of *M. merluccius* from 2.593 to 10.746 mg/kg ww throughout the study period. The mean concentration of As in the muscle tissue of *M. barbatus* and *M. merluccius* for the entire period was 19.689 mg/kg ww and 8.356 mg/kg ww, respectively (Table 2). The As concentrations in the muscle tissues of *M. barbatus* and *M. merluccius* were significantly higher in the coastal part of the open sea compared with the Bay. Differences in As concentrations in the fish samples depended on the sampling season (spring/autumn), with higher As concentrations in both fish species studied during the autumn sampling period. In 2019, higher concentrations of As were detected in the muscle tissue of *M. merluccius*, while higher concentrations of As were measured in *M. barbatus* sampled during 2020.

On the other hand, the Hg concentration in *M. barbatus* muscle tissue ranged from 0.181 to 0.801 mg/kg ww, with a mean concentration of 0.497 mg/kg ww, and in *M. merluccius* samples from 0.055 to 0.222 mg/kg ww, with a mean concentration of 0.153 mg/kg ww (Table 2). The Hg concentrations in the spring were higher in the muscle tissue of *M. barbatus* from the Bay than from coastal part of the open sea. Higher concentrations of Hg were detected in *M. barbatus* from the Bay, i.e., *M. merluccius* sampled in the coastal part of the open sea. There are no significant differences in Hg concentration in *M. barbatus* and *M. merluccius* samples between the examined periods (2019–2020).

In addition, the Pb concentrations in the muscle tissue of *M. barbatus* and *M. merluccius* were low throughout the study. The Pb concentration was higher in *M. barbatus* sampled in the Bay in the autumn than in the other time periods and sampling areas, and it was also higher than the Pb concentration in the muscle tissue of *M. merluccius.*

**Figure 2:**
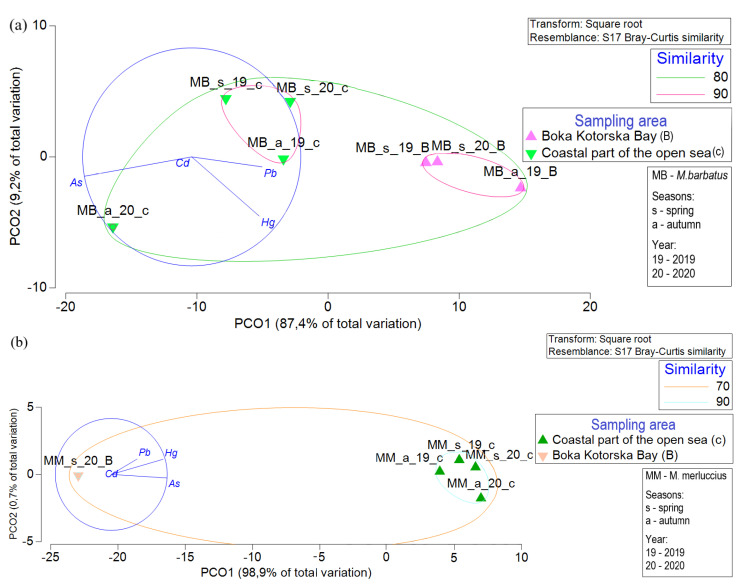
Principal coordinate analysis (PCO) performed to evaluate similarities according to the TE concentrations in (**a**) *M. barbatus* and (**b**) *M. merluccius* based on the different area of sampling, seasons, and year.

Figure 2 shows a graphical representation of TE concentrations in different fish species. The results of the PCO performed on the data collected in this study (Figure 2) confirm the results presented in Table 2. The PCO shows that two factors (PCO1 and PCO2) explain 96.6% of total variance for *M. barbatus* (Figure 2a) and 99.6% for *M. merluccius* (Figure 2b). There were significant differences between the concentrations of selected TEs sampled in different studied areas and between different fish species (PERMANOVA, Monte Carlo test, *p* < 0.05).

### 3.2. Health Risk Assessment

The THQ values of individual elements, as well as HI values due to the consumption of fish from Boka Kotorska Bay and the coastal part of the open sea, as well as the mean values in fish from the Montenegrin coast in general, based on the obtained results for these two areas during the study period, are presented in Table 3. THQ values for Pb and Cd were lower than 1 for both sampled fish species in both studied areas. THQ values for As were higher than 1 for both sampled fish species in both studied areas, while THQ values for Hg were higher than 1 only for *M. barbatus* sampled in Boka Kotorska Bay.

The concentrations of all studied TEs, and therefore THQ and HI values, were higher in the samples of *M. barbatus* than in the samples of *M. merluccius*. High THQ values for As were recorded in *M. barbatus* from the coastal part of the open sea, being 21.606 mg/kg∙day. High THQ values for As and Hg were also recorded in *M. barbatus* from the Boka Kotorska Bay, with 9.472 mg/kg∙day and 1.744 mg/kg∙day, respectively (Table 3). On the other hand, THQ values for all studied TEs in *M. merluccius* were less than 1, except for As, which was 2.1417 mg/kg∙day from Boka Kotorska Bay and 8.1667 mg/kg∙day from the coastal part of the open sea, as shown in Table 3. The values for the hazard index (HI), calculated as the sum of THQ for each element, were above 1 for both studied fish species in both studied areas (Table 3), which means that the exposed population could be exposed to health risks.

In this study, the dietary exposure to TEs through the consumption of *M. barbatus* and *M. merluccius* in the studied areas was evaluated using the estimated daily intake (EDI) and compared with the recommended daily dietary allowance (mg/day) by JECFA (2009), as shown in Table 4. The results presented in Table 4 show that Cd and Pb have the lowest daily intake, while Hg and As have the highest daily intake. The results of the EDI revealed that values for the examined fish samples were below the recommended levels for Pb and Cd and above the recommended As values for both fish species and the recommended Hg values for *M. barbatus*.

## 4. Discussion

The differences in the concentrations of the studied TE in fish depending on different seasons, areas, and years of sampling were not consistent. Such results are to be expected to some degree because, unlike shellfish, these are migratory species that move, feed, and live in different habitats, which may result in the uneven distribution and uptake of TEs depending on various factors observed (biotic and abiotic). In this context, Alamdar et al. (2017) point out that higher concentrations of TEs have been observed in benthic species compared to benthopelagic and/or pelagic fish species [37], which in turn implies that TEs are significantly more abundant in marine sediments than in seawater [38].

In general, the differences in the concentration of studied TEs in fish in relation to the sampling area are the result of natural factors such as geographical, morphological, pedological and hydrogeological differences between the two studied areas, but also various anthropogenic influences [17].

Both studied fish species contained high concentrations of As. The mean As concentrations in the muscle tissues of *M. barbatus* (19.689 ± 10.25 mg/kg ww) and *M. merluccius* (8.356 ± 3.28 mg/kg ww) were lower than the values reported from Italy [38,39] and higher than the literature data for the same fish species from the Adriatic and Mediterranean Sea (Table 5). As is a widespread metalloid that occurs naturally in the environment, especially in rocks and sediments, which could explain the lower prevalence of As in a benthic-pelagic fish species such as *M. merluccius* compared to a benthic fish species such as *M. barbatus* [40,41,42,43]. High concentrations of As in fish from the Adriatic Sea were previously reported by Bilandžić et al. (2011) and Perugini et al. (2013) [17,18]. In a study from the coast of Norway, As content was analyzed in different fish species fillets [44]. The authors reported that the content of As varied highly between species, (from 0.3 to 110 mg/kg ww) [44], which is a very large range compared to the range of As reported in the present study. Additionally, apart from marine fish, high concentrations of As have been recorded in freshwater fish [4]. Fattorini et al. (2008) believe that the differences in As concentration depending on the sampling area may be due to the influence of seawater salinity on the modulation of TE accumulation [45]. For As compounds in fish or other seafood, no maximum permissible levels have yet been established [30]. High As concentrations were detected in many fish, with the highest content of this element deposited in muscle tissues [46,47].

The mean Hg concentration in *M. barbatus* (0.497 ± 0.233 mg/kg ww) is comparable to data from Italy, the Adriatic Sea, and higher than Hg concentrations reported in Croatia, the Adriatic Sea, and Italy, Libya, and Spain, the Mediterranean Sea, for the same fish species (Table 5). The mean Hg concentration in *M. merluccius* (0.153 ± 0.06 mg/kg ww) was similar to or lower than the Hg concentrations reported in the Adriatic and Mediterranean Seas for the same fish species (Table 5). *M. barbatus* accumulated more Hg than *M. merluccius*, which is consistent with the idea that bottom-feeding fish (benthic species) tend to accumulate high levels of this TE compared with pelagic fish species [49]. The mean Hg levels in the studied fish in both observed areas and sampling periods, according to the EU legislation [30], are below the maximum permissible value levels for MeHg, which is similar to the level established for the total Hg for fish products and muscle tissues (0.5 mg/kg ww and 1.00 mg/kg ww for certain pelagic and benthopelagic fish species, such as *M. barbatus*) [30].

Compared to previous studies, the mean Pb concentration in this study was generally lower than or similar to that in muscle tissues of *M. barbatus* and *M. merluccius* from the Adriatic and Mediterranean Seas (Table 5). The maximum permissible levels for lead (0.3 mg/kg ww) established by EU legislation [30] are lower than the recorded mean values of Pb in the studied fish, which means that the level of Pb in all studied fish species is within the limits acceptable for human consumption.

The measured mean Cd concentration in the muscle tissues of all studied fish species was below 0.02 mg/kg ww and below the maximum permissible value for fish samples, which is given as 0.05 mg/kg ww by EU legislation [30]. The mean Cd concentration in the present study was low (below the detection limit) and comparable to values reported in the literature: 0.001–0.6 mg/kg ww for *M. barbatus* and 0.01–0.3 mg/kg ww for *M. merluccius* from the Adriatic and Mediterranean Sea (Table 5).

To assess human health risk associated with As, Hg, Pb, and Cd through the consumption of two studied fish species, the EDI, THQ, and HI were calculated. The THQ and EDI values showed that the intake rate of Pb and Cd were within the safe level for both studied fish species. The THQ values for Hg were only above 1 in *M. barbatus* from Boka Kotorska Bay, while the EDI values showed that the intake rate of Hg in *M. barbatus* was not within the safe level. In contrast, the THQ and EDI values for *M. merluccius* showed that the intake rate of Hg was within the safe level. The THQ and EDI values for As were high for both studied fish species, indicating that the intake rate of As was not within the safe level. HI values based on four TEs were high in both studied fish species, implying that human health may be at risk.

Although the results for Pb and Cd in this study were below the limits of permissible concentrations, Pb and Cd are non-essential, toxic elements that can be present in high concentrations in fish and could pose serious threats to both the health of the species themselves and to human health through consumption of seafood [57], so continuous monitoring is required.

It was concluded that As and Hg could pose a potential health risk to the population through the consumption of fish from the study region. Toxic inorganic As can accumulate in marine organisms and convert to less toxic organic As such as arsenobetaine [42]. The major As compound found in the majority of marine organisms, especially in fish, is nontoxic [4,58,59]. Many authors have noted that marine organisms have high concentrations of As [2,23,60,61], but most of this element is present in its organic fraction (arsenobetaine), which is nontoxic to human health [62]. Arsenobetaine is metabolically inert and nontoxic and is rapidly excreted in urine in humans [23]. Concentrations of organic As in marine organisms are directly proportional to total As concentrations [63]. Zhang et al. (2012) and Moxness Reksten et al. (2021) reported that the concentrations of inorganic As in fish account for 1–3% of the total As concentrations [16,64]. Based on the above, it is difficult to make a correct human health risk assessment of As from the consumption of the studied fish, since the percentage of inorganic As in fish is quite low or even negligible [4]. Higher values of Hg have been found in the sediment of the Adriatic Sea [65,66,67], so its increased value in samples of *M. barbatus* from Montenegrin coast is not surprising.

Hg is a non-essential and one of the most toxic TEs, and exposure to high concentrations of this element could have various consequences for biodiversity and human health, so it is important to monitor its concentration in the environment [41]. Additionally, Al-Busaidi et al. (2011) reported that the most significant source of Hg in humans’ diet is fish [68].

## 5. Conclusions

The results of this study provide important data on: marine environment pollution; the abundance of TEs in studied fish on the Montenegrin coast and differences in the concentration of examined TEs in relation to the different species of fish examined, different areas, season, and year of sampling. Additionally, the results of this study provide important data on the health risk to humans associated with exposure to TEs through the consumption of the studied fish species from the Montenegrin coast. The potential health risk assessments based on EDI and THQ values calculated for As and Hg recommend the moderate consumption of the studied fish species, while there are no consumption limits based on the amount of Pb and Cd in fish muscles. Considering our findings regarding TEs in fish, as well as the fact that fish is increasingly consumed in Montenegro, further investigation and monitoring are necessary to maximize consumer health protection. The present study provided a baseline and important contributions to future risk/benefit assessments for fish consumers in Montenegro. Similar studies need to be conducted on a regular basis to monitor the evolution of TE levels in all food products and to assess whether there is a potential health risk to consumers.

## Figures and Tables

**Table 1:** Comparison of certified and measured concentrations of TE in the muscle tissues of the studied fish species (mg/kg ww) in certified reference materials (IAEA 407, 436).

Trace Elements	Certified	Measured	Recovery (%)	Certified	Measured	Recovery (%)
	IAEA 407			IAEA 436		
Hg	0.222	0.223	100.4	/	/	/
Pb	0.12	0.12	100.0	/	/	/
As	12.6	12.5	99.2	1.98	1.97	99.5
Cd	0.189	0.187	98.9	0.052	0.052	100.0

/—CRM IAEA 407 was used for all studied TEs, while CRM IAEA 436 was used for As and Cd.

**Table 2:** TE concentrations (mg/kg ww) in muscle tissues of the studied fish species.

Species	Sampling Period	Sampling Area	Concentration of Trace Elements			
			As	Hg	Pb	Cd
*M. barbatus*	Spring 19	Boka Kotorska Bay	12.489	0.744	0.037	<0.02
		Coastal part of the open sea	23.520	0.181	0.033	<0.02
	Autumn 19	Boka Kotorska Bay	8.572	0.548	0.053	<0.02
		Coastal part of the open sea	21.606	0.547	0.034	<0.02
	Spring 20	Boka Kotorska Bay	13.021	0.801	0.023	<0.02
		Coastal part of the open sea	19.113	0.252	<0.02	<0.02
	Autumn 20	Coastal part of the open sea	39.501	0.408	<0.02	<0.02
	Mean value		19.689 ± 10.25	0.497 ± 0.233	0.031 ± 0.01	<0.02
*M. merluccius*	Spring 19	Coastal part of the open sea	9.522	0.176	0.034	<0.02
	Autumn 19	Coastal part of the open sea	9.016	0.156	0.025	<0.02
	Spring 20	Boka Kotorska Bay	2.593	0.055	<0.02	<0.02
		Coastal part of the open sea	9,901	0.222	<0.02	<0.02
	Autumn 20	Coastal part of the open sea	10.746	0.146	0.025	<0.02
	Mean value		8.356 ± 3.28	0.153 ± 0.06	0.025 ± 0.01	<0.02

**Table 3:** THQ and HI values for the individual TEs in different fish species and in the study area for fish consumers.

Species	Trace Elements	THQ		
		Boka Kotorska Bay	Coastal Part of the Open Sea	Montenegrin Coast
*M. barbatus*	As	9.472	21.61	16.84
	Hg	1.744	0.867	1.240
	Pb	0.038	0.018	0.020
	Cd	0.050	0.050	0.050
**HI**		11.304	22.545	18.15
*M. merluccius*	As	2.142	8.167	7.086
	Hg	0.137	0.437	0.382
	Pb	0.010	0.017	0.016
	Cd	0.050	0.050	0.050
**HI**		2.339	8.671	7.534

**Table 4:** Comparison of EDI of TEs of the studied fish species with the recommended daily dietary allowances.

Species	Trace Elements	EDI			Recommended Daily Dietary Allowance of JECFA [36]
		Boka Kotorska Bay	Coastal Part of the Open Sea	Montenegrin Coast	
*M. barbatus*	As	2.842	6.482	5.053	0.13
	Hg	0.174	0.087	0.124	0.03
	Pb	0.009	0.007	0.008	0.21
	Cd	0.005	0.005	0.005	0.06
*M. merluccius*	As	0.643	2.450	2.126	0.13
	Hg	0.014	0.044	0.038	0.03
	Pb	0.005	0.006	0.006	0.21
	Cd	0.005	0.005	0.005	0.06

**Table 5:** Comparison of TE concentrations in muscle of *M. barbatus* and *M. merluccius* found in this study and previous studies from the Adriatic and Mediterranean Seas, expressed in mg/kg ww.

Species	Sampling Site	TEs				Reference
		As	Hg	Pb	Cd	
*M. barbatus*	Montenegro (Adriatic Sea)	19.689	0.497	0.031	<0.02	Present study
	Croatia (Adriatic Sea)	5.91	0.06	0.02	0.002	[18]
	Italy (Adriatic Sea)	59.91	0.48	0.05	0.07	[38]
	Italy (Adriatic Sea)	/	0.43	0.10	0.08	[48]
	Italy (Adriatic Sea)	/	0.55	0.06	0.02	[49]
	Italy (Adriatic Sea)	/	0.49	/	/	[50]
	Italy (Mediterranean Sea)	9.477	0.054	0.006	0.002	[51]
	Italy (Mediterranean Sea)	12.09	0.18	0.05	<0.001	[23]
	Italy (Mediterranean Sea)	27.01	0.027	0.04	0.001	[39]
	Libya (Mediterranean Sea)	/	0.07	0.19	0.6	[52]
	Spain (Mediterranean Sea)	15.05	0.09	0.05	0.001	[53]
*M. merluccius*	Montenegro (Adriatic Sea)	8.356	0.153	0.02	<0.02	Present study
	Montenegro (Adriatic Sea)	/	/	0.25	/	[54]
	Montenegro (Adriatic Sea)	/	/	<0.1	<0.2	[55]
	Croatia (Adriatic Sea)	4.38	0.295	<0.05	<0.01	[56]
	Italy (Adriatic Sea)	38.70	0.59	0.03	0.06	[38]
	Italy (Adriatic Sea)	/	0.12	0.04	0.30	[49]
	Italy (Adriatic Sea)	/	0.18	/	/	[50]
	Italy (Mediterranean Sea)	8.75	0.15	0.03	<0.001	[23]

/—In the presented studies, analyzes of the selected TEs were not performed.

## Data Availability

The data are available from the corresponding author.
